# Lessons learned from adapting a remote area health placement from physical to virtual: a COVID-19-driven innovation

**DOI:** 10.5116/ijme.61b3.56ee

**Published:** 2021-12-31

**Authors:** Donna B. Mak, Kylie Russell, Dylan Griffiths, Daniel L. Vujcich, Roger Strasser

**Affiliations:** 1 School of Medicine, The University of Notre Dame Australia, Fremantle, Australia; 2 School of Population Health, Curtin University, Bentley, Australia; 3 TeHuataki Waiora School of Health, The University of Waikato, New Zealand

**Keywords:** Community-oriented partnership, community engagement, social capital, medical education

## Abstract

**Objectives:**

To investigate the acceptability and the effectiveness of a virtual adaptation of a well-established, mandatory, community-based pre-clinical remote area health placement in which medical students learn about the social and environmental determinants of health in remote Australia; and make recommendations to guide the delivery of future learning experiences.

**Methods:**

A mixed-methods convergent design was used. All 99 students, 36 placement hosts and 10 staff were invited to complete an online survey and 27(27%), 12(33%) and 10(100%), respectively, contributed data.  Qualitative data were collected via semi-structured interviews from four students, four hosts and six staff. Survey data were analysed using descriptive statistics (frequency and percentage) and open-ended responses summarised to provide supporting contextual evidence. Interview transcripts were analysed and coded independently, then corroborated to identify and summarise common themes using thematic analysis.

**Results:**

Survey and interview data indicated that the virtual placement was acceptable to students and hosts and enabled students to achieve intended learning objectives.   Virtual activities enabled students and hosts to develop authentic, genuine interpersonal relationships, which in turn were facilitated when hosts and students had practiced videoconferencing beforehand with good high-speed internet connections via mobile devices. Pastoral care and access to IT support were essential.

**Conclusions:**

Virtual placements can be used in combination with and are an option for students and hosts who cannot attend/courses that cannot fund physical placements. Careful design and further research is required to ensure that virtual placements enable "head, heart and hands" learning and do not create/reinforce inequities.

## Introduction

There is a growing body of literature concerning the impact of the coronavirus disease 2019 (COVID-19) pandemic on medical education. To date, much of the focus of the literature has been on the adaption of traditional classroom methods to virtual formats. Comparatively, little attention has been given to experiential learning methods, such as onsite placements. For instance, a recent systematic review of studies examining the application and effectiveness of virtual medical education found only four examples of the use of digital technology to deliver experiential learning during the pandemic.^1^ These were: a letter to the editor outlining a digital clinical placement;^2^ a description of how general practice clinical attachments were adapted in one medical school;^3^ a brief report of a small survey study (n=14) of students undertaking virtual ward rounds;^4^ and a brief report presenting survey results of a small (n=6) virtual clerkship program for internal medicine.^5^

Medical educators must continue to build this body of literature by sharing the methods, results, and lessons learnt from their attempts to adapt experiential learning methods in the context of the current pandemic. Medical students have expressed concerns about practical skill development due to their inability to participate in placements,^6^ and it has been argued that students (including international students) are losing opportunities for cross-cultural learning through place-based immersion.^7^ More guidance is needed to assist educators in providing a greater range of learning experiences to medical students in the context of continuing outbreaks of COVID-19.

In this article, we present the results from an adaptation of a mandatory, non-clinical placement program delivered by the University of Notre Dame School of Medicine's Fremantle campus in the state of Western Australia which occupies one third of the continent's land area and has a population of 2.6 million.^8^ The Program was established in 2005 with a view to contributing to the School's mission of graduating "doctors to serve in areas of unmet need, specifically in the country's vast remote and rural areas".^9^ In the Program, all first and second-year students (about 100 in each class) live with and/or undertake non-clinical activities with hosts in rural (first-year students) and remote (second-year students) areas of Western Australia to learn first-hand about the social and environmental determinants of health in remote Australia.^10^ The remote Kimberley region in far north Western Australia has hosted the second year placement every August since 2006. One of the most sparsely populated parts of the world, the Kimberley has a population of 36,000 spread over 420,000 square kilometres (almost 1.5 times the size of Italy).^11^ A recently published evaluation of the placement's perceived long-term impact on graduates and placement hosts indicated that the Program validated pre-existing interest in, or positively influenced graduates' attitudes towards, rural practice and fostered empathy and responsiveness when caring for rural patients in both urban and rural health services.^12^ Placement hosts unanimously supported the Program and contributed social capital to ensure its sustainability.^12^

In March 2020, the placement's instigator and academic coordinator (DBM) realised that COVID-19-related travel restrictions to prevent COVID-19 transmission to remote Aboriginal communities would preclude second-year students from undertaking a physical Kimberley placement. Over the next three months, university staff, the Shire of Derby West Kimberley, community-based organisations and previous placement hosts collaborated to develop a virtual experience to enable students to meet the same learning objectives. In July 2020, students undertook pre-placement learning activities delivered by videoconferencing (VC) technology, followed in August 2020 by a five-day virtual Kimberley placement comprising: (1) daily VC interactions between Perth-based students and Kimberley-based hosts; (2) the exchange of 'getting to know you' mail packages between students and hosts containing non-perishable items symbolising their life and home; and (3) daily recreational or experiential activities in Perth to enable students to develop a greater appreciation of Kimberley life through activities suggested by their hosts (e.g., visiting the Kimberley section of Perth's botanical garden, listening to music or relevant podcasts, watching online video content or reading). During the placement, students and hosts were 'visited' virtually by a university staff member and could access IT and pastoral support. See Appendix A, Table 1  for a detailed timetable of the preparatory, placement and post-placement learning activities.

The aim of this study was to:

    ·     document the acceptability of the virtual adaptation of the placement to students, staff and hosts;

    ·     assess the effectiveness of the virtual placement in enabling students to meet core learning outcomes associated with the traditional physical placement;

    ·     make recommendations to guide the delivery of future learning experiences for medical students in both pandemic- and post-pandemic contexts

## Methods

This descriptive study was undertaken using a mixed-method convergent design involving data from participant surveys and semi-structured in-depth interviews. This supported a pragmatic worldview centred on a real-world problem needing practical solutions.^13^

Two weeks after the completion of the virtual Kimberley placement, the school's quality assurance officer (DG) sent an email to medical students who had undertaken the placement, virtual placement hosts and university staff inviting them to participate in an online survey for internal quality assurance processes. The student and host surveys were identical to previously validated surveys used to evaluate student and hosts experiences of this placement with the omission of questions regarding travel and accommodation. The staff survey was adapted from one developed for use in a published evaluation of the first year rural health placement.^14^ All three surveys are included in Appendix B. To maximise response rates, three email reminders were sent, and the survey was closed four weeks after the Program's completion.

At the end of each survey, participants were provided with a research project participant information sheet and asked if they would consent to their survey responses being included in this study and participate in a one-on-one telephone or videoconference semi-structured interview; interview questions are included in Appendix B.

Forty-two of 99 (42%) of students participated in the evaluation survey, and 64% (27/42) of these consented for their survey data to be used for research purposes (Table 1). Two-thirds (24/36) of community hosts participated in the evaluation survey, and one half (12/24) of these consented to provide their survey data for research purposes (Table 1).

**Table 1:** Research participants

Participants/ respondents	Student placements n (%)^*^	Student survey respondents n (%)	Student research participants n (%)	Placement hosts n (%)^*^	Host survey respondents n (%)^*^	Host research participants n (%)
Total	99	42	27	36	24	12
Placement type						
Pastoral station	11 (11)	6 (14 )	3 (11 )	5 (14 )	1 (4 )	0 (0 )
School in town	3 (3 )	3 (7 )	2 (7 )	2 (6 )	2 (8 )	2 (17 )
School in an Aboriginal community	6 (6 )	1 (2 )	1 (4 )	2 (6 )	1 (4 )	1 (8 )
Government department, e.g., Dept of Parks and Wildlife	35 (35 )	7 (17 )	3 (11 )	11 (31 )	6 (25 )	4 (33 )
Other, e.g., art gallery, private citizens from a range of backgrounds including accountant, truck driver, health care workers	44 (44 )	25 (60 )	18 (67 )	16 (44 )	14 (58 )	5 (42 )

*Percentages do not add to 100% due to rounding

Of 10 University staff who participated in the placement, all consented to provide their survey data for research purposes. All four students, four hosts and six staff who agreed to be interviewed were interviewed between September and October 2020. In order to minimise the risk of bias, neither of the interviewers had participated in the placement. KR (who interviewed students) was not involved in the delivery of the medical degree and did not have a pre-existing relationship with the student respondents. DV is an external academic and was not a colleague of the staff he interviewed. Interviews were conducted and audio-recorded and ranged from 30 – 60 minutes in length.

Analysis of the survey and interview data was conducted independently by DG (survey) and DLV and KR (interview) before being corroborated to identify and summarise any common themes. Survey data were analysed using descriptive statistics (frequency and percentage), with qualitative aspects from the open-ended responses summarised to provide supporting contextual evidence.

Interview data were transcribed by an independent provider and then analysed and coded using Braun and Clark's six phases of thematic analysis.^15^ Initial coding and theme identification were undertaken by DLV and KR and reviewed and confirmed by the wider research team. It is important to note that one of the staff interviewees (staff interview 2) was a co-author of this article (DBM). It was considered important to include this interviewee's perspective given her close involvement in both the virtual placement that is the subject of this article and past physical placements. Data triangulation suggested that data saturation had been achieved in that the themes emerging from the qualitative interviews were broadly consistent with the survey results.^16^ While efforts were made to interview divergent cases (e.g., the minority of survey participants who reported negative experience of the placement), they declined to participate in interviews, and the findings ought, therefore, to be read in light of this limitation.

To reduce the potential of social acceptability bias and conflicts of interest due to unequal working relationships, data collection and analysis were conducted by members of the research team that were not involved in student educational outcomes related to the Program. Demographic data such as gender and age were not included in surveys or interviews to ensure de-identification of research participants, given the small numbers involved in the placement. Ethics approval was provided by the University of Notre Dame's Human Research Ethics Committee.

## Results

### Students' experiences and learning

Both the survey and interview data showed that students felt welcomed by their individual hosts and the broader communities. Almost all (92.6%) student survey respondents agreed (37.0%) or strongly agreed (55.6%) that their host made them feel welcome, with the remaining 7.4% neither agreeing nor disagreeing. This statement, "My host put in a lot of effort to arrange different activities for us each day which I think made my placement experience more positive as I enjoyed all our sessions thoroughly" is typical of student survey responses about the highlight of their virtual placement. All of the student interviewees expressed their gratitude to the community for their level of engagement and desire to share their experiences with them.

"I think the effort and kind of enthusiasm that the towns put into the videos was really good." (student interview 1)

The 'getting to know' you packages facilitated rapport between students and hosts.

"I think people found the exchange of packages with the host to be very strange, but then when it actually happens, everyone could see the value in it… it turned out to be a really nice touch and a really good way to break the ice and actually make that connection." (student interview 4)

The quality of interactions between students and hosts was, in large part, dependent on individual backgrounds, personalities and skills. One student whose host was of a similar age and also had a young family commented that this

"meant that we already have a lot in common, and I think that helped." (student interview 4)

Some students enhanced their virtual experience with Kimberley-relevant physical experiences in Perth, including sitting under the boab trees at the state's botanical garden (student interview 4) and visiting other "culturally significant sites" (student interview 2). Some of these experiences were suggested by hosts "Aunty so-and-so says to do this. I'm going to go and do this and see how I like it" (student interview1).

The majority of students reported that formal educational resources including the 'Living on medicine' PBL case, preparatory activities and formal group learning sessions helped them to learn from their placement experience and meet their learning objectives (Appendix A, Table 2). However, students found the volume and duration of online activities excessive, with only 37% (n=10) of students agreeing/strongly agreeing that the 'length of the placement was appropriate', while 44.4% (n=12) either disagreed or strongly disagreed. The sentiment was reflected in the interviews with students, all of whom commented on the experience of 'Zoom overload':

"There was too much Zoom I can't even remember some of the things that were discussed or talked about… it was just too much." (student interview 3)

While students valued their conversations with hosts and the formal group learning sessions, they realised that it was impossible to replicate a physical Kimberley placement experience through a virtual medium and physical co-location and sharing of physical experiences would enable deeper connection and understanding:

"We just had conversations… it was interesting hearing about her life [but] it was quite disengaging…" (student interview3),

"You can't, like cook a meal with someone, you can't walk around with someone… you miss all of those relatable and personal moments" (student interview1)

and

"I personally am not a huge fan of [rodeos] … but I know it's important for the town, but when you are far removed from it all you're seeing is the things that you see negatively because you aren't within that positive atmosphere." (student interview 2)

Despite these limitations, the majority of students who participated in the survey reported that the virtual placement: (1) prompted them to question some of their beliefs and opinions (59.3%); (2) led them to reflect on attitudes to health and values associated with treatment/management (74%); (3) provided them with a better understanding of 'remoteness' (51.8%), the health issues facing people (77.8%) and Aboriginal people (77.8%) living in remote areas, and the diversity of remote cultures and languages (81.5%); and (4) generated more interest in working with Aboriginal people (59.2%)(full results shown in Appendix A, Table 3).

Pre-existing interest in rural and remote health was high, with almost two-thirds of students have applied for optional rural/remote placements, investigated other curricular opportunities to learn about rural/remote medical practice, or investigated working rurally/remotely post-graduation before the virtual Kimberley placement. Following the virtual Kimberley placement an additional 25.9% of students intended to participate in extra-curricular opportunities in the medical curriculum to learn about rural and remote area practice (chi-square 4.8, df=1, p=0.3). There were statistically insignificant increases in the proportions of students who intended/were pleased to be based rurally for the whole of their third year, investigate other curricular opportunities to learn about rural/remote medical practice, investigate living and to work in rural and remote areas after graduation, and investigate working in rural and remote areas after graduation on a fly-in-fly-out / drive-in-drive-out short term locum basis (Appendix A, Table 4).

### Hosts' experiences

The vast majority of hosts reported that that the placement provided an authentic experience for students to learn about the life, community and living in the Kimberley; that the placement was worthwhile for their family/organisation/business and that they were satisfied with their interactions with students and University staff (Appendix A, Table 5).

Where technology permitted (e.g., good wireless internet connections), some hosts attempted to provide more immersive experiences by taking students on 'virtual' tours:

"We spent an hour a day where they came home with me virtually to see a typical Broome kind of house and environment… a little bit of the neighbourhood and then we went to the areas around Broome." (host interview 1)

Similar to students, hosts reported the absence of physical co-location and sharing of physical experiences to be the main limitation of the virtual placement:

"you don't get to smell the smells, you don't see the kids running around … you just don't have that incidental stuff happening that is equally as important." (host interview 1)

Despite these limitations, host interview data consistently emphasised the value of a virtual placement in circumstances in which physical placements are not possible:

"there's pluses and minuses with this...but it certainly wasn't a pale watered-down experience." (host interview 3)

Of the six host survey respondents (50%) who had hosted students on a physical placement, three agreed or strongly agreed that student engagement during the virtual placement was similar to that during previous physical placements and three disagreed or strongly disagreed.   

The only virtual host interviewed who had hosted students in previous physical placements was of the view that, in some respects, the virtual format improved the quality of their interactions:

"I feel like I probably even knew them better than the guys that we'd had up here in the past." (host interview 3)

Two-thirds of hosts (8/12) surveyed reported that they would be willing to host students virtually or in-person in future years. The remaining one third answered unsure, with the most frequent reason for their response was that it would depend on the situation regarding COVID-19 and/or if they are still in the Kimberley.

### Staff perceptions

Nine out of ten staff reported that the engagement of students with placement hosts and community members was authentic, and 80% reported that it would assist students in caring for patients from remote locations in metropolitan health services (Appendix A, Table 6).

Similar to students and hosts, staff acknowledged the importance of physical co-location:

"it’s almost impossible to recreate that experience digitally… I’ve been to that rodeo, it’s as much the smells and the wandering around and just seeing the locals enjoying it” (staff interview 4)

and,

“The tour of the Bungarun leprosarium in person was one of the more powerful things I’ve experienced with the students, and many students [were] moved to tears … that sense of feet on graves actually out there, it’s very powerful … [the virtual tour is] just not the same as being there.” (staff interview 6)

Staff identified that an unintended consequence of the virtual format was that people need a good internet connection and feel comfortable using videoconferencing technology in order to be a host:

“the people who agreed to be the hosts were tech savvy”(staff interview 1)

and

“basically cowboys don’t Zoom … this particular trip … was prejudiced probably against remote Aboriginal communities and cattle stations and very much biased towards town people.” (staff interview 5)

Furthermore, the verbal, conversational nature of the virtual placement, in contrast to the hands-on, kinaesthetic nature of physical placements also led to unintended consequences:

“some hosts inherently they’re good talkers and they take well to Zoom, and then there are other hosts that they’re lovely people, but the way to learn with them is to be with them physically … they become a bit awkward on Zoom.” (staff interview 2)

Technological issues such as poor picture and sound quality and rough editing of video tours were perceived by some staff as a barrier to student engagement and learning:

“I would ideally budget having some sort of production crew do something that’s a lot slicker... If it looks rough and you can’t hear it properly, and you know, fades in and out, and the camera’s moving too quickly and stuff, I think people get turned off.” (staff interview 1)

However, other staff considered the ‘low production value’ of some content as a strength in that it helped to add authenticity:

“It was certainly not polished, but perhaps it worked because of that … [T]here was a clear element of the community were going to a lot of trouble to welcome the students as though they were there live … there was a beautiful element of authenticity to that that worked really well.” (staff interview 4)

Staff identified unintended positive consequences of the virtual placement, including virtual placements being an option for students who cannot attend physical placements because of health- or family-related reasons and virtual tours enabling large numbers of students to “visit” a health service and meet a wide variety of staff while maintaining COVID-19-safety:

“I think that’s a real bonus that those people aren’t excluded anymore” (staff interview 2), and “bridging distance” (staff interview 5) with videoconferencing technology:

“One of the things that we learnt was that the Kimberley people can participate in the pre and post events via video conferencing … it collapses distances around all those sorts of thing … we’ve got an opportunity to even better engage with them and have them as more equal partners who are participating in the full … program, not just the bit [in which] we’re actually up there.” (staff interview 4)

## Discussion

Survey and interview data from students, hosts and staff indicate that despite the limitations associated with delivering an immersive, experiential placement using a virtual format and the impossibility of replicating a physical Kimberley placement experience via VC, the virtual placement enabled students to achieve the intended learning outcomes. The placement prompted students to question some of their beliefs and opinions; (2) led them to reflect on attitudes to health and values associated with treatment/management; (3) provided them with a better understanding of remoteness, the health issues facing people, including Aboriginal people in remote areas; (4) generated more interest in working with Aboriginal people and (5) encouraged students to participate in rural extra-curricular activities. Hosts were satisfied with the opportunity to engage with students via VC.

Although only 27 of the 42 students (64%) and 12/24 hosts (50%) who completed the survey agreed for their survey data to be used in this research project, their responses were comparable with those of all students and hosts who completed the survey. The proportion of students whose data were included in the research is representative of those who undertook placements on pastoral stations, town schools and schools in an Aboriginal community. However, students placed with government departments were under-represented in the research sample, and those in ‘other’ placements (e.g., art gallery, private citizens) were over-represented. For hosts, the proportion of data included in the research for schools in an Aboriginal community, a government department and ‘other’ is representative of all placement participants, whereas pastoralists were under-represented and town schools were over-represented.

The student survey used in this research aligns closely with that administered to students following previous physical Kimberley placements. Student satisfaction with placement-related teaching and learning resources (Appendix A, Table 2 ) was higher in 2020 compared with 2019.  Comparison of student survey research data with that collected following the 2019 physical placement indicates that the virtual and physical placements were comparable in enabling students to achieve the placement’s intended learning objectives about the social and environmental determinants of health and issues associated with access to health care in remote Australia (Appendix A, Table 3).

One of the key motivating factors for implementing the virtual placement was to demonstrate the School of Medicine’s unwavering commitment to its mission of graduating 'doctors to serve in areas of unmet need, specifically in the country’s vast remote and rural areas' and continue its 15-year partnership with the Kimberley community.^9^ This is particularly important during the COVID-19 pandemic because health care provision in rural and remote Australia is highly reliant on short-term locums based in urban centres and overseas trained workers. COVID-19 related inter-and intra-national border closures resulted in severe health workforce shortages across much of rural and remote Australia.^17^

These health workforce shortages limit access not only to health care, but also to clinical placement opportunities, highlighting the importance of partnering with communities for student teaching. A recent systematic review of rural workforce retention showed that community engagement and support are key factors to health worker retention.^18^ Continuing the Kimberley placement in virtual format in 2020 enabled the School to maintain engagement with the Kimberley community as demonstrated by our students being welcomed back by placement hosts during the 2021 physical placement and safeguards our students’ opportunity for a remote area learning experience if demand for clinical placements in remote Australia outstrips supply.

While virtual placements are not a perfect substitute for physical placements, our study shows that they can be a valuable alternative where barriers to physical placements exist. Aside from barriers related to the COVID-19 pandemic, our virtual placement model may also be suitable in response to other barriers, including personal circumstances (e.g., students being able to travel due to family commitments) or broader structural barriers such as availability of resources. For non-medical health professions, the largest component of the health workforce,^19^ limited funding, placement models, accommodation, and a lack of supervisory staff form significant rural clinical placement barriers,^20^^,^^21^ a substantial concern given the World Health Organisation recommendation to improve rural health through embedded curricula and immersions.^22^ Investment in non-traditional and multidisciplinary placements is complex, and despite adding further capacity, growing student numbers to meet health workforce demands impedes overall capacity.^23^ Engagement with a virtual immersion may provide an innovative solution for curricula developers and practitioners to support widespread student learning and engagement with isolated and in-need populations.  Community engagement and participation in regional development, as demonstrated in this study, is a key element to support and grow rural training.^24^

Where circumstances necessitate the use of a virtual placement model, careful consideration must be given to recognising and, where possible, addressing its limitations. We offer the following recommendations which build on the existing literature:

It is well reported that virtual teaching can create and reinforce inequities in under-resourced settings.^25^ In the Kimberley placement, many of the most geographically isolated hosts and communities were unable to participate because of limited or no access to internet connections suitable for VC. Consequently, the virtual placement involved many more town-based hosts than previous physical placements and very few pastoral stations and remote Aboriginal communities. This experience of poor internet connectivity is not limited to the Kimberley region.^26^ In the absence of substantial government investment in the communications infrastructure, virtual placement coordinators may need to develop workaround solutions such as providing some participants with portable wireless internet devices, or devising complementary educational experiences to compensate for the exclusion of certain groups and perspectives.

Learning is facilitated through the integration of three domains: “cognitive (head), affective (heart) and psychomotor (hands)”.^27^ The physical Kimberley placement embodies “head, heart and hands learning” with students living and working alongside their hosts. By contrast, the virtual adaption was able to capture much of the “head”, some of the “heart” and very little of the “hands” learning. While students valued the structured learning sessions (“head”) and expressed much appreciation for host and community efforts to share their lives (“heart”), they missed the lived experience that students who undertook previous physical placements reported as being invaluable for learning how to care for patients from rural and remote areas. The loss of a sense of “presence” has been reported as a barrier to learning in other areas of medical education during the COVID-19 pandemic, and efforts to recreate immersive, sensory experiences are being explored, including through the use of augmented reality technology.^28^ Virtual placement coordinators must be cognisant of the limitations of the online teaching medium and should seek innovative ways of approximating the sense of “presence”. In the Kimberley placement, students acknowledged the utility of the “getting to know you” packages and relevant physical experiences in Perth (e.g., visiting the Kimberley section of the botanical garden). While such innovations have value, it must be recognised that they can never adequately replicate the physical experience of working alongside people in a rural and remote area context. Additional training may therefore be needed to give future doctors the skills and confidence to effectively communicate with and relate to people living in rural and remote areas. 

Student respondents shared their experience of “Zoom fatigue” which has been identified as a barrier to communication and learning in other studies.^1^^,^^29^ Virtual placement experiences would be improved by scheduling more regular breaks between online sessions, limiting the duration of VC teaching blocks, and maintaining as much on-campus face-to-face small group teaching as possible. The use of purpose-made, pre-recorded footage of adequate sound and visual quality interspersed with live interaction with health professionals was also identified as a valuable way of maintaining student interest and engagement.

As learning to live with COVID-19 becomes the “new normal” with lifting of travel restrictions and increasing COVD-19 vaccination coverage of vulnerable populations, we need to consider the benefits of a virtual placement carefully in protecting vulnerable communities from potential COVD-19 transmission and enabling participation by students who cannot travel versus the longer-term risks of future doctors being less well equipped to communicate with and relate to remote-area Australians because the virtual format is less effective at delivering “heart and hands” learning.

In 2021 we incorporated virtual elements into the physical placement to enhance learning and reduce COVID-19 transmission risk, e.g., physical health service tours were replaced by short pre-recorded videos including interviews with a variety of clinical and managerial staff followed by a facilitated videoconference discussion with the senior medical officer, at least one person from the Kimberley was included via videoconference in all pre-placement preparatory activities, and students who were unable to travel undertook a virtual placement instead of being excluded from the placement experience as in previous years because they would have received a medical exemption from the placement.

We have summarised the advantages and disadvantages of the virtual Kimberley placement in Box 1 and our tips for adapting a successful physical placement to a virtual format in Box 2.

**Box 1:** Advantages and disadvantages of virtual placement

Advantages	Disadvantages
· Increase opportunities for students and hosts to participate	· Create and reinforce inequities in under-resourced settings
· Favours “head” learning	· Harder to create opportunities for “heart” and “hands” learning
· Can be used in combination with physical placement to enhance learning	· Cannot replicate a physical experience
· Minimises physical risk	· “Zoom fatigue”

## Conclusions

Evaluation using a mixed-method convergent design involving data from surveys and semi-structured in-depth interviews of students and hosts indicated that the virtual placement enabled students to achieve intended learning outcomes and hosts to provide an authentic experience for students to learn about living in a remote area. Not just “better than nothing”, the virtual placement confirmed other pandemic experiences that what was previously considered “impossible” is both “possible” and positive. Virtual remote health placements have educational value and may be used to

complement physical placements when public health, personal or financial constraints prevent students from traveling.  Careful design and further research is required to ensure that virtual placements enable “heart” and ‘hands” as well as “head” learning, and do not create/reinforce inequities.

**Box 2:** Tips for adapting a successful physical placement to a virtual format

Communicate. Involve all program partners and stakeholders from the beginning to ensure participation.
Rehearse. Do a test run with each host beforehand to ensure their internet connection and VC skills can support their participation indoors and outdoors.
Enable as much “heart” and “hands” learning as is possible in a virtual format. Schedule activities that encourage and enable students and hosts to develop authentic, genuine interpersonal relationships.
Recognise the limitations of technology. It is better for students to interact with hosts who have good internet access and videoconference (VC) skills than set the Program up for failure by trying to include locations with poor internet access and hosts who are uncomfortable with VC.
Beware “Zoom fatigue”. Limit VC duration, schedule frequent breaks and a mix of online and face-to-face activities.
Care for students and hosts. Pastoral care and logistical support is as necessary for virtual as for physical placements. Ensure access to IT support. Schedule ‘visits’ by supervising academics and debriefings.
Be brave and creative. Necessity is the mother of invention and a pandemic provides the impetus and freedom for new approaches to old problems to be trialed.

### Acknowledgments

The Kimberley remote area health placement is funded by the Australian Government's Rural Health Multidisciplinary Training Program. The authors thank the Shires, communities and placement hosts whose support and participation are the key to the placement's sustainability and positive influence on Australia's future doctors.

### Conflict of Interest

The authors declare that they have no conflict of interest.

## Supplementary material

Supplementary file 1 Appendix A. Table 1 - 6

Supplementary file 2 Appendix B. Surveys and Interview Questions
