# Supplementary material for: Lessons learned from adapting a remote area health placement from physical to virtual: a COVID-19-driven innovation
Source: Int J Med Educ. 2021 Dec 31;12:274–99. doi: 10.5116/ijme.61b3.56ee (PMC12928721; doi:10.5116/ijme.61b3.56ee)
Supplement: Supplementary file 1 — Appendix A. Table 1 - 6 [file ijme-12-274-S1.pdf]

Appendix A

Table 1. Program Timetable

Week	Date	Activity
8	Monday 9th March	• First briefing 10:30-11:15 (45mins)
21	Monday 8th June	• Virtual Kimberley Placement briefing 13:00-13:30
26	Monday 13th July	• Film: *Yajilarra* and tutor-facilitated reflection using de Bono's 6 thinking hats 10:00-11:00<br>• Films: *The healing sounds of the Bungarun Orchestra* + video by WinunNgari Aboriginal Corporation and reflection using de Bono's 6 thinking hats 11:00-12:30
	Friday 17th – Monday 20th July	• Prepare and post the 'getting to know you' package for your host.
27	Monday20th July (Fremantle)	• 1030 - 1120 Debate 1 Doctors should treat everyone the same regardless of who they are or where they come from.<br>• 1130 - 1220 Symposium 1 "History and health" Self-determination is a major theme in history and in public health. Give examples from the Kimberley of the relationship between history, past and current public health practices. From your readings and experiences, suggest some ways forward.
28	Monday 27th July	• 1030-1120 Symposium 2: Occupational and recreational health and safety during Kimberley placements.<br>• 1130-1220 Symposium 3. "What we learn from history" Federal government commitments to Oxfam Australia's "Close the Gap" on Indigenous health inequality have had major impacts on some Indigenous people and possibly relatively little impact on others. Drawing on the most recent Close the Gap report tabled in Federal parliament, use the published views of Aboriginal authors and representatives of Aboriginal organisations to clearly articulate some of the positives and negatives of these policy interventions over the last 11 years. The presenting groups should make three to four substantiated recommendations based on their readings.
29	Monday 3rd August	• 1130-1220 Symposium 4 Population study of the West Kimberley
	Thursday 6th August	• 1330-1500 Preparatory Personal and Professional Development tutorial
	Friday 7th August	• 1600 Shire welcome including welcome to country<br>• 1630-1800 Derby town tour including sunset over the mudflats and high tide at the jetty
	Saturday 8th August	• 0800-0830 Derby town tour to show low tide at jetty<br>• 830-1030 problem-based learning (*before* the virtual Kimberley placement) tutorial 1 Living on Medicine<br>• 1030-1130 Derby Drs and Rural Clinical School student panel: "Working out Bush"<br>• 1145-1230 Renal Disease in Kimberley Aboriginal people lecture –<br>• 1330-1415 Derby Hospital Tour<br>• 1415-1500 Derby Aboriginal Health Service Tour
	Sunday 9th August	• 0900-1130 (including 15-20min break) Aboriginal Cultural and Linguistic Workshop<br>• 1230-1330 Bungarun Leprosarium visit<br>• 1330-1500 Derby rodeo
30*	Monday 10th August	• First student-host videoconference - Introduction & opening of 'getting to know you' packages (1hr)<br>• Virtual Kimberley experience 1 (2hrs)
	Tuesday 11th August	• 0800-1015 Dr Kim Isaacs - an introduction to place, context and settings<br>• 2nd student-host videoconference - family, home, garden (1hr)<br>• Virtual Kimberley experience 2 (2hrs)
	Wednesday12th August	• 0800-1015 Bart Pigram - history, culture and place<br>• 3rd student-host videoconference – work / daily routine (1hr)<br>• Virtual Kimberley experience 3 (2hrs)
	Thursday 13th August	• 0800-1015 Nyamba Buru Yawuru - building opportunities, growing language and growing *Mabuliyan*<br>• 4th student-host videoconference - What makes me happy and keeps me well (1hr)<br>• Virtual Kimberley experience 4 (2hrs)

	Friday 14th August	• 5th student-host videoconference – Something special I'd like to share with you as a farewell gift (1hr)<br>• Virtual Kimberley experience 5 (2hrs)<br>• 1500-1545 Post placement Body Parts debriefing via Zoom breakout groups<br>• 1600-1700 Debate 2 Recruiting and retaining rural health staff – what works better? Carrots or sticks?
31	Monday 17th August	• 1030-1120 Debate 3 Telehealth, remote consultations, FIFO/DIDO and other technological solutions are the future of remote area health care<br>• 1130-1220 Symposium 5 '*We acknowledge the traditional owners'* Aboriginal people, including elders, community leaders, professionals and workers, have key roles in maintaining and improving the health and wellbeing of Aboriginal and non-Aboriginal Australians. Reflect on the roles and contributions of Aboriginal people and Aboriginal community-controlled organisations that you have encountered during your Kimberley placement. What do they offer that is valuable and how might medical practitioners benefit from working with Aboriginal people/organisations? What are some of the challenges faced by medical practitioners and Aboriginal organisations when they try to work together and how have these been addressed?<br>• Prepare for evidence-based medicine tutorial 1330-1500<br>• Evidence based medicine tutorial (in PBL groups) 1500-1630
	Thursday 20th August	• 1330-1500 Post-placement Personal and Professional Development tutorial<br>• PBL tutorial 2 1500-1700
32	Monday 24th August	• PBL tutorial 3 1330-1430

*During week 30, all student-host groups were visited at least once by an academic supervisor at a pre-arranged time and individual students and hosts were able to contact a university staff member during office hours for information technology and pastoral support.

Table 2. Student responses to Likert survey items on the effectiveness of teaching and learning resources (n=27)

Statements	Strongly disagree (%)	Disagree (%)	Neither agree nor disagree (%)	Agree (%)	Strongly Agree (%)
The 'Living on medicine' problem focused my learning on the placement	0	2 (7.4)	9 (33.3)	16 (59.3)	0
The preparatory resources and activities (explanatory notes, population study, briefings, films, debates, symposia, CD tutorial) helped me to prepare for the placement.	0 (0.0)	4 (14.8)	5 (18.5)	17 (63.0)	1 (3.7)
The preparatory resources and activities helped me to learn from my placement and meet my PBL learning objectives.	0 (0.0)	6 (22.2)	7 (25.9)	12 (44.4)	2 (7.4)
The placement resources and activities (health service and Bungarun site visit, talk by Derby Drs, cultural and linguistic orientation) helped me to learn from my placement experience and meet my PBL learning objectives.	0 (0.0)	3 (11.1)	2 (7.4)	19 (70.4)	3 (11.1)
The post-placement resources and activities (debates/symposia, CD tutorial, EBM tutorial, portfolio reflection) helped me to learn from my placement and meet my PBL learning objectives.	1 (3.7)	6 (22.2)	8 (29.6)	12 (44.4)	0 (0.0)
I met most learning objectives using the resources provided before, during and after the placement	1 (3.7)	5 (18.5)	7 (25.9)	13 (48.1)	1 (3.7)

Table 3. Student responses, impact of the virtual Kimberley placement (n=27)

Statements	Strongly disagree (%)	Disagree (%)	Neither agree nor disagree (%)	Agree (%)	Strongly Agree (%)
As a result of the virtual Kimberley remote area health placement I have:					
been prompted to question some of my beliefs and opinions	0 (0.0)	4 (14.8)	7 (25.9)	15 (55.6)	1 (3.7)
reflected on attitudes to health and values associated with treatment/management that I may previously have taken for granted	0 (0.0)	2 (7.4)	5 (18.5)	17 (63.0)	3 (11.1)
a better understanding of the health issues of people living in remote areas	1 (3.7)	2 (7.4)	3 (11.1)	17 (63.0)	4 (14.8)
a better understanding of the health issues of Aboriginal people living in remote areas	1 (3.7)	3 (11.1)	2 (7.4)	17 (63.0)	4 (14.8)
a better understanding of the diversity of cultures and languages in remote Western Australia	0 (0.0)	3 (11.1)	2 (7.4)	17 (63.0)	5 (18.5)
a better appreciation of 'remoteness', the magnitude of distance, the issues of communication, the isolation from others and from services	3 (11.1)	1((3.7)	9 (33.3)	7 (25.9)	7 (25.9)
more interest in working with Aboriginal people	0 (0.0)	1 (3.7)	10 (37.0)	10 (37.0)	6 (22.2)
an enhanced interest in rural/remote area practice	0 (0.0)	4 (14.8)	10 (37.0)	8 (29.6)	5 (18.5)

Table 4. Student responses *before*, and intentions *following*, the virtual Kimberley placement to investigate/pursue opportunities in rural/remote health (n=27)

Statements	Students reporting they had taken this action *before* the virtual Kimberley placement, n (%)	Students reporting this intention *following* the virtual Kimberley placement, n (%)	Students reporting not having this intention *following* the virtual Kimberley placement, n (%)	Students reporting being unsure about this intention following the virtual Kimberley placement, n (%)
Apply to participate in Broome learning on country (BLOC)*	15 (55.5)	Not applicable because BLOC was timetabled to occur before the virtual Kimberley placement		
Apply to (before placement)/are pleased to or wish to (following placement) study in the Rural Clinical School WA in your third year*	18 (66.7)	20 (74.1)	4 (14.8)	3 (11.1)
Investigate other opportunities in the medical curriculum to learn about rural and remote area practice	14 (51.9)	20 (74.1)	5 (18.5)	2 (7.4)
Participate in extra-curricular opportunities to learn about rural and remote area practice	7 (25.9)	15 (55.5%)	8 (29.6)	4 (14.8)
Investigate living and working in rural and remote areas after graduation	17 (63.0)	19 (70.4)	3 (11.1)	5 (18.5)
Investigate working in rural and remote areas after graduation on a fly-in-fly-out / drive-in-drive-out short term locum basis	9 (33.3)	15 (55.5)	8 (29.6)	4 (14.8)
Participate in a new activity/ interest or re-engage in a previous activity/interest not relate directly to medicine. If yes, specify	6 (22.2)	6 (22.2)	9 (33.3)	12 (44.4)

*Optional curricular rural/remote placement

Table 5. Host responses on impact of the virtual placement (n=12)

Statements	Strongly disagree (%)	Disagree (%)	Neither agree nor disagree (%)	Agree (%)	Strongly Agree (%)
Engagement of medical students during the virtual placement was similar to that during previous physical placements*	1 (16.7)	2 (33.3)	0 (0.0)	2 (33.3)	1 (16.7)
The virtual placement provided an authentic experience for students to learn about my life, my community and living in the Kimberley	1 (8.3)	0 (0.0)	2 (16.6)	6 (50.0)	3 (25.0)
The placement was worthwhile for my business/organisation/ family	0 (0.0)	0 (0.0)	1 (8.3)	7 (58.3)	4 (33.3)
I was satisfied with the liaison between staff from the school of medicine, university of Notre Dame and my organisation/business/ family before the placement	0 (0.0)	0 (0.0)	0 (0.0)	3 (25.0)	9 (75.0)
I was satisfied with the IT support provided by STAFF from the School of Medicine, University of Notre Dame to prepare for my organisation/business/ family for the virtual placement	0 (0.0)	0 (0.0)	2 (16.7)	4 (33.3)	6 (50.0)
I was given sufficient notice of when to send in the 'getting to know you' package.	0 (0.0)	0 (0.0)	1 (8.3	4 (33.3)	7 (58.3)
I was satisfied with the supervision of students by university of Notre Dame staff during the placement	0 (0.0)	0 (0.0)	2 (16.7)	9 (75.0)	1 (8.3)

Table 6. Staff responses to Likert survey items on impact of the virtual placement (n=10)

Statements	Yes (%)	No (%)	Unsure (%)
Do you perceive that the students' interaction with the local community members was authentic, and of value to understanding the context of remote area living?	9 (90.0)	0 (0.0)	1 (10.0)
Do you perceive that the Virtual Kimberley Remote Area Health Placement will provide value to students caring for patients from remote locations in metropolitan health services?	8 (80.0)	0 (0.0)	2 (20.0)
Do you think it is important to have learning outcomes to underpin this type of learning activity?	10 (100.0)	0 (0.0)	0 (0.0)
