# Supplementary material for: Lessons learned from adapting a remote area health placement from physical to virtual: a COVID-19-driven innovation
Source: Int J Med Educ. 2021 Dec 31;12:274–99. doi: 10.5116/ijme.61b3.56ee (PMC12928721; doi:10.5116/ijme.61b3.56ee)
Supplement: Supplementary file 2 — Appendix B. Surveys and Interview Questions [file ijme-12-274-S2.pdf]

Appendix B

Surveys and Interview Questions

MEDI6200 Virtual Kimberley Remote Area Health Placement 2020 – Student Survey

Dear MEDI6200 student

Your survey responses will help the school evaluate this virtual placement to improve teaching for the benefit of future students. The School would also like to use these data for a research project so we can publish our findings to share what we have learned with the broader academic community and policy-makers. If you would prefer your responses NOT to be used in the research, you will have the opportunity to opt-out at the end of this survey.

*denotes compulsory question

*1. Learning Resources

	Strongly Disagree	Disagree	Neutral	Agree	Strongly Agree
The 'Living on medicine' problem focused my learning on the placement					
The preparatory resources and activities (explanatory notes, population study, briefings films, debates, symposia, tutorial) helped me to prepare for the placement.					
The preparatory resources and activities helped me to learn from my placement and meet my learning objectives.					
The preparatory resources and activities helped to alleviate any anxiety associated with the virtual placement.					
The placement resources and activities (health service and Bungarun site visit, talk by Derby Drs, cultural and linguistic orientation) helped me to learn from my placement experience and meet my PBL learning objectives.					
The post-placement resources and activities (debates/symposia, tutorial, portfolio reflection) helped me to learn from my placement and meet my PBL learning objectives.					
I met most learning objectives using the resources provided before, during and after the placement.					

*2. As a result of the virtual Kimberley remote area health placement I have:

	Strongly Disagree	Disagree	Neutral	Agree	Strongly Agree
a better understanding of the health issues of people living in remote areas.					
a better understanding of the health issues of Aboriginal people living in remote areas.					
a better understanding of the diversity of cultures and languages in remote Western Australia.					
reflected on attitudes to health and values associated with treatment/management that I may previously have taken for granted.					
more interest in working with Aboriginal people.					
a better appreciation of 'remoteness', the magnitude of distance, the issues of communication, the isolation from others and from services.					
had a positive experience of what it would be like to live in rural and remote areas.					
been prompted to question some of my beliefs and opinions.					
an enhanced interest in rural/remote area practice					

Comments

*3. Please select the type of location of your placement from the list below

Pastoral station	School (Aboriginal community)
School	Government department/agency (e.g. Dept. of Parks and Wildlife)
Aboriginal community	Non-government/community services(e.g. community radio)
Family/household	Business (specify)
Other (please specify)	

4. Please state the name of your location (optional).

5. What do you think you contributed to your placement host/organisation?

6. What advice would you give a future student participating in a virtual placement with this host/organisation?

*7. Organisation

	Strongly Disagree	Disagree	Neutral	Agree	Strongly Agree	Not Applicable
The length of the placement was appropriate						
I felt safe (in a personal sense) during the placement.						
I was given sufficient notice of when and what to send in the 'getting to know you' package.						
Arrangements for academic activities (e.g. cultural and linguistic orientation, PBL, Drs panel discussion) were appropriate						
My placement host made me feel welcome						

Comments

*8. What for you was the highlight of the Virtual Kimberley Remote Area Health Placement?

9. Is there anything you think should have been done differently before, during or after the Virtual Kimberley Remote Area Health Placement?

10. Do you have any suggestions on how the Virtual Kimberley Remote Area Health Placement could be improved in the future for the benefit of the students and/or the communities where you stayed?

*11. Between completing the Wheatbelt placement and starting the virtual Kimberley placement did you do any of the following (Please tick all that apply).

	Yes	No
Apply to participate in the Broome - learning on country (BLOC) placement		
Apply to study in the Rural Clinical School in your third year		
Investigate other opportunities in the medical curriculum to learn about rural and remote area medical practice		
Participate in extra-curricular opportunities in the medical curriculum to learn about rural and remote area practice		
Investigate living and working in rural and remote areas after graduation		
Investigate working in rural and remote areas after graduation on a Fly In Fly Out/Drive In Drive Out short term locum basis		
Investigate working in rural and remote areas before graduation in a non-medical job on weekends or during university holidays		
Participate in a new activity/ interest or re-engage in a previous activity/interest not related directly to medicine, if yes, specify		

12. *Following the Virtual Kimberley Remote Area Health Placement do you intend to do any of the following (Please tick all that apply)

	Yes	No	Not sure
Investigate other opportunities in the medical curriculum to learn about rural and remote area medical practice			
Participate in extra-curricular opportunities in the medical curriculum to learn about rural and remote area practice			
Are pleased you will be, or wish you could, study in the Rural Clinical School			
Investigate living and working in rural and remote areas after graduation			
Investigate working in rural and remote areas after graduation on a Fly In Fly Out/Drive In Drive Out short term locum basis			
Investigate working in rural and remote areas before graduation in a non-medical job on weekends or during university holidays			
Participate in a new activity/ interest or re-engage in a previous activity/interest not related directly to medicine, if yes, specify			

13. If you would like to participate in a 20 minute telephone interview to provide the School with more information about your experience of the 2020 virtual Kimberley placement, please provide your name, email address and telephone number. Please note that to maintain your anonymity, your response to this question will be separated from your responses to previous questions.

Name	
Email	
Telephone	

Thank you for completing this survey and, most importantly, for participating in the 2020 virtual Kimberley placement and providing feedback. Your responses will be used for internal evaluation of the placement.

Interview questions: Students

1. Tell me a little about your virtual placement. *Prompt with below topic guide as required*
    a. Tell me about the placement host?
    b. Was it a positive experience?
    c. Were you nervous or shy, were they… did this impact the experience?
    d. Were there other factors that influenced the experience?
    e. Have you, or do you think you will, remain in contact?
2. Why do you believe the School of Medicine has the Kimberley Placement? *Prompt with below topic guide as required*
    a. Student learning
    b. Understanding of rural living
    c. Understanding of rural health resources/access
    d. Future workplace option
    e. Aboriginal health
3. What do you believe the benefits of the Program are for you and your fellow students? If there was no benefit, why? (they may be a rural student). *Prompt with below topic guide as required*
    a. Rural and remote living?
    b. Rural and remote health care?
    c. Future workplace/student placement opportunities?
4. How do you feel you can incorporate what you have learnt from this virtual Kimberley experience into your practice as a future clinical student and doctor?
5. Has this Program sparked any other interests as a result of your interactions? (may be related or not related to medicine)
6. What do you think your placement host gained from the virtual experience?
7. If you attended the Wheatbelt CEW, how did this compare, being in person vs virtual?
    a. If you were unable to attend the CEW, do you believe this virtual placement provided you with an opportunity to participate, given that you may have otherwise missed this experience? Why is this important/not important to you?
8. Is there anything about the Program (content/format/timing/platform) that you believed did not support your learning or engagement?
9. Is there anything that you would like to add? Prompt with below topic guide as required
    a. Any suggestions for improvement?

MEDI6200 Virtual Kimberley Remote Area Health Placement – Placement Host Survey

Dear placement host

Your survey responses will help the school evaluate this virtual placement to improve teaching. The School would also like to use these data for a research project so we can publish our findings to share what we have learned with the broader academic community and policy-makers. If you would prefer your responses NOT to be used in the research, you will have the opportunity to opt out at the end of this survey.

*denotes compulsory question

*1. Please identify your placement type

Pastoral station	School (Aboriginal community)
School	Government department/agency (e.g. Dept. of Parks and Wildlife)
Aboriginal community	Non-government/community services(e.g. community radio)
Family/household	Business (specify)
Other (please specify)	

*2. Please identify your location

Broome	
Derby	
Fitzroy Crossing	
Other (please specify)	

*3. The number of students placed with you in 2020

*4. I was satisfied with the liaison between STAFF from the School of Medicine, University of Notre Dame and my business/organisation/family BEFORE the placement.

Strongly Disagree	Disagree	Neither Agree nor Disagree	Agree	Strongly Agree

*5. I was satisfied with the IT support provided by STAFF from the School of Medicine, University of Notre Dame to prepare my business/organisation/family for the virtual placement.

Strongly Disagree	Disagree	Neither Agree nor Disagree	Agree	Strongly Agree

*6. I was given sufficient notice of when and what to send in the 'getting to know you' package.

Strongly Disagree	Disagree	Neither Agree nor Disagree	Agree	Strongly Agree

*7. Was the timing of the placement (early August) appropriate?

Yes	
No – it should be earlier in the year	
No – it should be later in the year	

Reasons for alternate timing

*8. Was the length of the placement appropriate?

Yes	
No – too long	
No – too short	
Unsure	

Comment

*9. I was satisfied with the interaction between the MEDICAL STUDENTS and my business/organisation/family DURING the placement?

Yes	
No	
Unsure	

*10. Engagement of MEDICAL STUDENTS during the virtual placement was similar to that during previous physical placements

Strongly Disagree	Disagree	Neither Agree nor Disagree	Agree	Strongly Agree	Not Applicable – 1st time as a placement host

Comment

*11. The virtual placement provided an authentic experience for students to learn about my life, my community and living in the Kimberley.

Strongly Disagree	Disagree	Neither Agree nor Disagree	Agree	Strongly Agree

Please provide an example to explain your response.

*12. I was satisfied with the SUPERVISION of students by University of Notre Dame staff DURING the placement.

Strongly Disagree	Disagree	Neither Agree nor Disagree	Agree	Strongly Agree

Comment

*13. I appreciated being offered the opportunity to attend and observe medical student tutorials, lectures and panel discussions online even if I did not attend any of these activities.

Strongly Disagree	Disagree	Neither Agree nor Disagree	Agree	Strongly Agree

Comment

*14. Number of medical student tutorials, lectures and panel discussions that I observed

Comment

Q15 only for people who answered >=1 in Q14

*15. The medical student tutorials, lectures and panel discussions that I observed were engaging and informative.

Strongly Disagree	Disagree	Neither Agree nor Disagree	Agree	Strongly Agree

*16. I was satisfied with the LIAISON between staff from the School of Medicine, University of Notre Dame and my business/organisation /family DURING the placement.

Strongly Disagree	Disagree	Neither Agree nor Disagree	Agree	Strongly Agree

*17. The placement was worthwhile for my business/organisation/family

Strongly Disagree	Disagree	Neither Agree nor Disagree	Agree	Strongly Agree

*18. Would you like to host students next year?

Yes – physical and/or virtual placements	
Yes - virtual placement only	
Yes – physical placement only	
No	
Unsure	

Please provide reasons for your response

19. Do you have any suggestions on how the virtual placement could be improved for hosts and/or students?

20. The School is always looking for new placement hosts for medical students. If you would like to recommend a person or organisation please provide some contact details here and the School will contact them

21. If you would like to participate in a 20 minute telephone interview to provide the School with more information about your experience of the 2020 virtual Kimberley placement, please provide your name, email address and telephone number. Please note that to maintain your anonymity, your response to this question will be separated from your responses to previous questions.

Thank you for completing this survey and, most importantly, for participating in the 2020 virtual Kimberley placement and providing our future doctors with a deeper understanding of life in the Kimberley. Your support for this placement is invaluable to its success and is much appreciated.

Interview questions: Placement hosts

1. What was your involvement in the virtual Kimberley Placement?
2. Have you been involved before, in what capacity? If yes, how did the virtual experience compare with the physical experience? Were you able to achieve the same learning and engagement experience with your student/s. What was better, what was difficult to achieve, or not as good as you had hoped?
3. What do you believe the School of Medicine's purpose of the Kimberley Placement was? Do you feel this was achieved? *Prompt with below topic guide as required*
    a. Student learning
    b. Understanding of rural living
    c. Understanding of rural health resources/access
    d. Future workplace option
    e. Aboriginal health
4. What, if anything, about the virtual placement do you think could be changed to improve the experience for students and placement hosts?
5. What do you believe are the benefits of the Program to you, your local town/community?
6. What do you see as the benefits of the placement for students?
7. If travel restrictions remain in place, would you like to see the Program continue? Would you like to see the Program continue in addition to the traditional Program? Providing a choice of engagement for both hosts and students.
8. If travel restrictions are lifted, would you like to see the virtual Kimberley placement continue? If yes why, if no why?
9. Is there anything that you would like to add? *Prompt with below topic guide as required*
    a. Any suggestions for improvement?

MEDI6200 Virtual Kimberley Remote Area Health Placement – Staff Survey 2020

Dear colleague
Your survey responses will help the school evaluate this virtual placement to improve teaching. The School would also like to use these data for a research project so we can publish our findings to share what we have learned with the broader academic community and policy-makers. If you would prefer your responses NOT to be used in the research, you will have the opportunity to opt out at the end of this survey.

*denotes compulsory question

*1. Please indicate your level of employment

Academic – Permanent	
Academic – Sessional	
Academic – Volunteer based in the Kimberley	
General Staff	

*2. The Virtual Kimberley Remote Area Health Placement was well planned and I understood my role

Strongly Disagree	Disagree	Neither Agree nor Disagree	Agree	Strongly Agree

Comments

*3. Were you involved in the planning of the Virtual Kimberley Remote Area Health Placement?

Yes	
No	

*4. Do you think that the Virtual Kimberley Remote Area Health Placement was of an appropriate length?

Yes	
No, too short	
No, too long	
Not Sure	

Comments

*5. The Virtual Kimberley Remote Area Health Placement was of benefit to the placement hosts/organisations and their communities

Strongly Disagree	Disagree	Neither Agree nor Disagree	Agree	Strongly Agree

Comments

6. The placement hosts/organisations were well prepared for the Virtual Kimberley Remote Area Health Placement?

Strongly Disagree	Disagree	Neither Agree nor Disagree	Agree	Strongly Agree

*7. What do you believe were the key learning outcomes achieved by students participating in the Virtual Kimberley Remote Area Health Placement (List as many as you wish)?

*8. Do you think it is important to have learning outcomes to underpin this type of learning activity?

Yes	
No	
Not sure	

Comments

*9. What do you believe are the key benefits to students participating in the Virtual Kimberley Remote Area Health Placement (List as many as you wish)?

*10. Do you perceive that the students' interaction with the local community members was authentic, and of value to understanding the context of remote area living?

Yes	
No	
Not sure	
Not applicable in my role	

Comments

*11. Do you perceive that the Virtual Kimberley Remote Area Health Placement will provide value to students caring for patients from remote locations in metropolitan health services?

Yes	
No	
Not sure	

*12. Would you like to see the Virtual Kimberley Remote Area Health Placement continue?

Yes, if travel restrictions remain	
Yes, even if travel restrictions are lifted	
No	

*13. Which students do you believe should attend the Virtual Kimberley Remote Area Health Placement?

All students	
Only students who cannot participate in a physical placement (if a physical placement is possible)	
No students (this is not a worthwhile learning experience)	

Comments

14. If you would like to participate in a 20 minute telephone interview to provide the School with more information about your experience of the 2020 virtual Kimberley placement, please provide your name, email address and telephone number. Please note that to maintain your anonymity, your response to this question will be separated from your responses to previous questions.

Thank you for completing this survey and, most importantly, for participating in the 2020 virtual Kimberley placement and providing our future doctors with a deeper understanding of life in the Kimberley. Your support for this placement is invaluable to its success and is much appreciated.

Interview questions: Staff

1. What was your involvement in the virtual Kimberley Placement?

2. Have you participated in the Kimberley Placement in the past? If yes, how do you compare the physical and the virtual experiences?
   a. What do you believe the benefits of the Program are for students, placement hosts, wider community and the School of Medicine? If there was no benefit, why? Be sure to cover all groups before moving on
3. Do you believe that the virtual placement engaged students with rural and remote issues and their future practice as a doctor?
4. What do you see as the strengths of the virtual Kimberley placement? Prompt future workplace/student opportunities, Aboriginal Health.
5. If travel restrictions remain in place, would you like to see the virtual Kimberley placement continue? If yes why, if no why?
6. If travel restrictions are lifted, would you like to see the virtual Kimberley placement continue? If yes why, if no why?
7. Would you like to see an extension of the virtual placement opportunity to other areas? Do you believe it should be provided to students unable to attend the Wheatbelt CEW, unsuccessful in their BLOC or RCSWA application?
8. What changes, if any, could benefit the Program?

9. Is there anything else that you would like to add? *Prompt with below topic guide as required*

   a. Any suggestions for improvement?
